# Predictors of Treatment Success Following Pulsed Radiofrequency of the Lumbar Dorsal Root Ganglion: A Multivariable Prediction Model with Clinical Nomogram

**DOI:** 10.1007/s11916-026-01544-x

**Published:** 2026-08-24

**Authors:** Matteo Luigi Giuseppe Leoni, Marco Mercieri, Sandra Magnoni, Giuliano Lo Bianco, Jamal Hasoon, Omar Viswanath, Giacomo Farì, Giuseppina Chelo, Giustino Varrassi

**Affiliations:** 1 Department of Medical and Surgical Sciences and Translational Medicine, Sapienza University of Rome, Rome, 00189 Italy; 2 Unit of Interventional Pain Management, Guglielmo da Saliceto Hospital, Piacenza, Italy; 3 Visionary International Board for Research and Analgesia (VIBRA), Fondazione Paolo Procacci, Rome, 00193 Italy; 4 Department of Medicine, Surgery and Pharmacy, University of Sassari, Sassari, 07100 Italy; 5 Anesthesiology and Pain Department, Foundation G. Giglio Cefalù, Palermo, 90015 Italy; 6 Department of Anesthesiology, Critical Care, and Pain Medicine, The University of Texas Health Science Center at Houston, Houston, TX USA; 7 Department of Experimental Medicine (Di.Me.S), University of Salento, Lecce, 73100 Italy; 8 Pain Unit, Anesthesia and General Intensive Care Unit, AOU Sassari, Sassari, Italy; 9 College of Medicine, University of Baghdad, Baghdad, Iraq

**Keywords:** Pulsed radiofrequency, Dorsal root ganglion, Chronic radicular pain, Prediction model, Nomogram, LASSO regression

## Abstract

**Background:**

Pulsed radiofrequency of the lumbar dorsal root ganglion (DRG-PRF) is a minimally invasive treatment for chronic radicular pain, but outcomes vary substantially and validated prediction models to guide patient selection are lacking. The aim of this study was to develop and internally validate a multivariable prediction model identifying patients most likely to achieve treatment success following lumbar DRG-PRF.

**Methods:**

This retrospective cohort study included 308 consecutive patients who underwent DRG-PRF for chronic lumbar radicular pain. Positive outcome was defined as ≥ 50% pain reduction on the Numerical Rating Scale at 6-month follow-up. Candidate prognostic variables were selected using Least Absolute Shrinkage and Selection Operator (LASSO) regression and included in the multivariable logistic regression analysis. Model performance was evaluated using area under the receiver operating characteristic curve (AUC-ROC) and calibration plots. Internal validation employed 10,000 bootstrap replications. A clinical nomogram was developed for bedside application.

**Results:**

Treatment success was achieved in 43.5% of patients (134/308). LASSO identified four independent predictors: baseline pain intensity (OR = 1.558, *p* = 0.013), daily morphine milligram equivalents (OR = 0.981, *p* < 0.001), pain duration (OR = 0.816, *p* = 0.008) and previous fusion/decompression surgery (OR = 0.315, *p* = 0.004). The model demonstrated moderate discrimination (AUC = 0.734, 95%CI:0.678–0.788), good calibration (Hosmer-Lemeshow *p* = 0.454), and robust internal validation (optimism-corrected AUC = 0.742). At optimal cutoff (0.478), sensitivity was 67.2% and specificity 69.0%.

**Conclusions:**

This validated prediction model incorporating opioid consumption, pain duration, baseline pain intensity, and surgical history enables individualized risk stratification for lumbar DRG-PRF. The accompanying nomogram facilitates clinical decision-making and patient counseling.

**Supplementary Information:**

The online version contains supplementary material available at 10.1007/s11916-026-01544-x.

## Introduction

Chronic low back pain with radicular symptoms remains a substantial clinical challenge, affecting an estimated 9.4% to 25% of the general population and representing a leading cause of disability worldwide [1]. When conservative management, including pharmacological therapy, physical rehabilitation, and interventional approaches such as epidural steroid injections, fails to achieve adequate symptom control, pulsed radiofrequency of the dorsal root ganglion (DRG-PRF) has emerged as a minimally invasive neuromodulatory option to provide short-term analgesia [2]. PRF delivers short bursts of radiofrequency current (typically 20 milliseconds at 2 Hz) while maintaining tissue temperature below 42 °C, thereby preserving neural architecture and analgesia is achieved through non-thermal mechanisms [3, 4].

Despite the analgesic mechanisms underlying PRF effects remain incompletely understood, studies show selective ultrastructural effects on C- and Aδ-fibers without injury to myelinated fibers [5], along with reduced c-Fos expression in the dorsal horn, indicating altered central nociceptive processing [6]. PRF also modulates local inflammatory mediators [3] and may enhance descending inhibitory pathways [7]. Additional work suggests transient modulation of neuronal excitability and glial activity, contributing to its sustained clinical effects [8]. These combined mechanisms likely explain the variability in patient response and highlight the importance of optimized patient selection. In fact, despite growing clinical utilization and mechanistic insights, treatment outcomes following lumbar DRG-PRF exhibit considerable variability, with reported success rates ranging from 32% to 87% across published series [9–12]. This substantial heterogeneity suggests that not all patients benefit equally from PRF intervention, yet reliable predictors of treatment success remain poorly defined. Current patient selection relies predominantly on diagnostic criteria (such as clinical presentation and confirmatory diagnostic blocks) and anatomical considerations, with limited integration of patient-specific characteristics that may influence treatment response [13, 14].

Several retrospective analyses have identified potential prognostic factors associated with the efficacy of DRG-PRF [15–17]. However, these observations have not yet been integrated into validated multivariable prediction models capable of supporting clinical decision-making. This lack of evidence-based prognostic tools represents a relevant gap in the field, as suboptimal patient selection may expose individuals to unnecessary procedural risks with limited therapeutic benefit, while conversely depriving suitable candidates of a potentially effective intervention. Therefore, the aim of this study was to develop and internally validate a multivariable prediction model for identifying patients most likely to achieve a positive clinical outcome following lumbar DRG-PRF.

## Methods

### Study Design and Patient Population

This retrospective cohort study was conducted at Interventional and Surgical Pain Unit Centre, Guglielmo da Saliceto Hospital, Piacenza, Italy, from May 2023 to September 2025. The study protocol was approved by the local Institutional Review Board (protocol number: 747/2023/DISP) and was performed in accordance with the principles of the Declaration of Helsinki. Due to the retrospective nature of the study, the requirement for informed consent was waived by the ethics committee. Consecutive patients with lumbar radicular pain who underwent DRG-PRF were identified from the internal database. The inclusion criteria included: (1) age ≥ 18 years; (2) chronic lumbar radicular pain (duration > 3 months) corresponding to L4, L5, or S1 nerve root distribution; (3) failure of conservative management including pharmacotherapy and physical therapy for at least 3 months; (4) positive response to diagnostic transforaminal nerve root block performed at least 4 weeks prior to PRF; (5) complete clinical and follow-up data available at 6-month assessment. Exclusion criteria were: (1) pregnancy; (2) active local or systemic infection; (3) coagulopathy or ongoing anticoagulant therapy that could not be safely discontinued; (4) presence of implanted cardiac devices or other active electronic implants; (5) previous spinal surgery at the target level within 6 months; (6) progressive neurological deficit or cauda equina syndrome requiring urgent surgical intervention; (7) spinal instability, severe central canal stenosis (< 10 mm anteroposterior diameter), or multilevel foraminal stenosis; (8) primary pain generator other than radiculopathy (e.g., sacroiliac joint pathology, facet joint arthropathy as primary pain source); (9) active malignancy or known metastatic disease to the spine; (10) uncontrolled psychiatric disorder or active substance abuse; and (11) ongoing litigation or compensation claims related to the pain condition.

## Data Collection

Electronic medical records were systematically reviewed to extract demographic, clinical, and procedural data. Baseline characteristics collected included: age, gender, body mass index (BMI), pain duration (years), previous lumbar spine surgery (categorized as no surgery, discectomy only, or fusion/decompression procedures), vertebral level(s) treated (L4, L5), number of levels treated (single vs. multiple), and medication use. Medication data were categorized as: (1) opioid analgesics: documented as current use (yes/no) and quantified as morphine milligram equivalents (MME) per day calculated according to standard conversion factors [18]; (2) gabapentinoids (gabapentin or pregabalin): documented as current use (yes/no).

Clinical outcome measures were collected at two time points: baseline (pre-procedure) and 6-month follow-up. The 6-month assessment was deliberately chosen to evaluate mid-term efficacy rather than early or short-term responses, as clinically meaningful evaluation of DRG-PRF should focus on sustained pain relief over time rather than transient early effects.

Pain intensity was evaluated using the Numerical Rating Scale (NRS), an 11-point scale ranging from 0 (no pain) to 10 (worst imaginable pain). A positive outcome was defined as achieving a ≥ 50% reduction in NRS pain score from baseline at the 6-month follow-up. All patients underwent diagnostic transforaminal nerve root block at least 4 weeks prior to DRG-PRF to confirm the pain generator. Patients were considered to have a positive diagnostic block if they reported ≥ 50% pain reduction for a duration consistent with the expected duration of local anesthetic effect (2–4 h) and could perform previously painful movements with reduced discomfort.

## DRG-PRF Procedure

DRG-PRF procedures were performed in a dedicated interventional pain management suite equipped with a C-arm fluoroscopy system. All procedures were conducted under strict aseptic conditions following established institutional protocols for infection prevention. The full procedural details of DRG-PRF have been described in our previously published work [19]. Briefly, the intervention was performed under fluoroscopic guidance with the patient in the prone position and standard cardiorespiratory monitoring. After sterile preparation and local anesthesia, a curved-tip 18G RF cannula was advanced into the dorsal-cranial quadrant of the intervertebral foramen. Correct positioning was confirmed through contrast injection, impedance measurements (< 400 Ω) and sensory–motor stimulation thresholds (sensory ≤ 0.4 V; motor ≥ 1.5× sensory). PRF was delivered at 45 V, 20 ms pulses at 2 Hz for 360 s while maintaining tissue temperature < 42 °C to avoid thermal neurodestruction. Patients were monitored post-procedure for 30–60 min and discharged with standard post-procedural instructions.

### Statistical Analysis

Continuous variables are presented as mean ± standard deviation (SD) and categorical variables as frequencies and percentages. Normal distribution of continuous variables was assessed using the Shapiro-Wilk test. Positive outcome was defined as a ≥ 50% reduction in NRS score at 6 months compared with baseline following DRG PRF. For baseline characteristics, comparisons between patients with and without positive outcomes were performed using independent samples t-tests for continuous variables and chi-square tests or Fisher’s exact test when indicated for categorical variables. To identify predictors of positive outcome, a Least Absolute Shrinkage and Selection Operator (LASSO) regression with 10-fold cross-validation was applied. The LASSO penalization was used to reduce overfitting by shrinking regression coefficients, with variables having non-zero coefficients at the optimal lambda value selected for inclusion in the final model. The optimal lambda parameter (λ) was chosen based on the minimum cross-validation error (lambda.min). Selected predictors from LASSO were then entered into a multivariable logistic regression model. Model performance was evaluated using the area under the receiver operating characteristic curve (AUC-ROC), calculated with 95% confidence intervals via 10,000 bootstrap iterations. The optimal probability cutoff was determined using Youden’s index, maximizing the sum of sensitivity and specificity. Calibration was assessed through calibration plots comparing predicted probabilities with observed proportions across deciles, complemented by the Hosmer-Lemeshow goodness-of-fit test. Multicollinearity was evaluated using variance inflation factors (VIF), with VIF < 5 considered acceptable. Internal validation was performed using bootstrap resampling (10,000 iterations) to estimate optimism-corrected performance metrics and assess model stability. A clinical nomogram was developed to facilitate bedside risk prediction.

Longitudinal analyses of pain (NRS scores) were conducted comparing baseline and 6-month assessments. All statistical tests were two-tailed, with p-values < 0.05 considered statistically significant. Missing data were handled using complete case analysis, as missing data rates were < 5% for all variables. Model reporting followed the Transparent Reporting of a multivariable prediction model for Individual Prognosis Or Diagnosis (TRIPOD) statement guidelines [20]. All analyses were performed using R v4.5.2 (R Foundation for Statistical Computing, Vienna, Austria; www.r-project.org).

## Results

A total of 308 patients who underwent lumbar DRG-PRF were included in this analysis. At 6-month follow-up, 134 patients (43.5%) achieved a positive outcome, while 174 patients (56.5%) did not meet this threshold. Baseline demographic and clinical characteristics stratified by outcome status are presented in Table 1.

**Table 1:** Baseline characteristics stratified by outcome

Characteristic	Negative outcome (*n* = 174)	Positive outcome (*n* = 134)	*p*-value
Age (years)	62.52 ± 14.43	61.01 ± 13.97	0.358
Male, n (%)	105 (60.3%)	81 (60.4%)	1.000
BMI (kg/m²)	26.11 ± 4.65	26.18 ± 4.16	0.890
Opioid use, n (%)	107 (61.5%)	53 (39.6%)	**< 0.001**
Gabapentinoids use, n (%)	110 (63.2%)	81 (60.4%)	0.705
MME (mg/day)	40.36 ± 42.40	17.04 ± 26.22	**< 0.001**
Previous surgery, n (%)	66 (37.9%)	33 (24.6%)	**0.018**
Surgery type, n (%)			**< 0.001**
– Discectomy	23 (13.2%)	23 (17.2%)	
– Fusion/Decompression	43 (24.7%)	10 (7.5%)	
Pain duration (years)	3.49 ± 1.63	2.88 ± 1.66	**0.001**
Level L5–S1, n (%)	74 (42.5%)	53 (39.6%)	0.682
Multiple levels, n (%)	78 (44.8%)	58 (43.3%)	0.877
NRS baseline	7.87 ± 0.70	8.04 ± 0.76	**0.049**

Patients who achieved a positive outcome differed markedly in both medication use and surgical history. Specifically, they exhibited significantly lower opioid consumption (mean MME: 17.04 vs. 40.36; *p* < 0.001) and a reduced overall prevalence of opioid use (39.6% vs. 61.5%; *p* < 0.001). Previous lumbar surgery was also less common among patients with a positive response after DRG-PRF (24.6% vs. 37.9%; *p* = 0.018), with a particularly lower rate of prior fusion or decompression procedures (7.5% vs. 24.7%; *p* < 0.001). In addition, patients with a positive outcome had a significantly shorter duration of pain symptoms (2.88 ± 1.66 vs. 3.49 ± 1.63 years; *p* = 0.001). Finally, patients who achieved a positive outcome also demonstrated slightly higher baseline pain intensity (NRS: 8.04 ± 0.76 vs. 7.87 ± 0.70; *p* = 0.049).

**Fig. 1:**
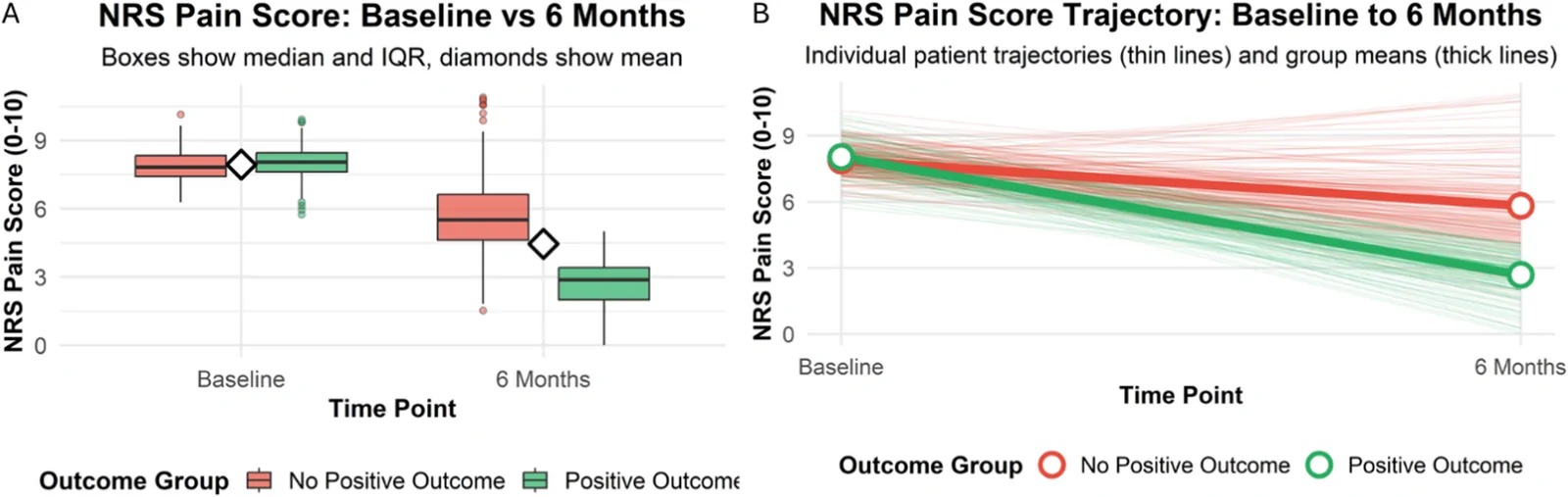
Changes in pain intensity from baseline to 6-month follow-up after DRG-PRF. **A** Boxplots of NRS pain scores at baseline and 6 months stratified by outcome group. Boxes represent the median and interquartile range (IQR), while diamonds indicate the mean values. Patients achieving a positive outcome (≥ 50% NRS reduction) showed a substantially greater decrease in pain compared with non-responders. **B** Individual pain score trajectories (thin lines) and group mean trajectories (thick lines) from baseline to 6 months, illustrating divergent pain evolution between patients with and without positive outcomes

Analysis of pain intensity from baseline to 6-month follow-up revealed a significant improvement. Global mean NRS decreased from 7.94 ± 0.73 at baseline to 4.46 ± 2.16 at 6 months, representing an overall reduction of 3.48 points (95% CI: 3.22–3.74, *p* < 0.001, Cohen’s d = − 1.514). Patients achieving positive outcomes experienced markedly greater pain reduction (− 5.34 ± 1.18 points, *p* < 0.001, d = − 4.537) compared to those without positive outcomes (− 2.05 ± 1.89 points, *p* < 0.001, d = − 1.085). This difference was statistically significant (mean difference = 3.13 points, 95% CI: 2.81–3.45, *p* < 0.001, Cohen’s d = 2.084), (Fig. 1A). Trajectory analysis demonstrated graphically the same divergent patterns between outcome groups (Fig. 1B).

### Correlation Analysis

**Fig. 2:**
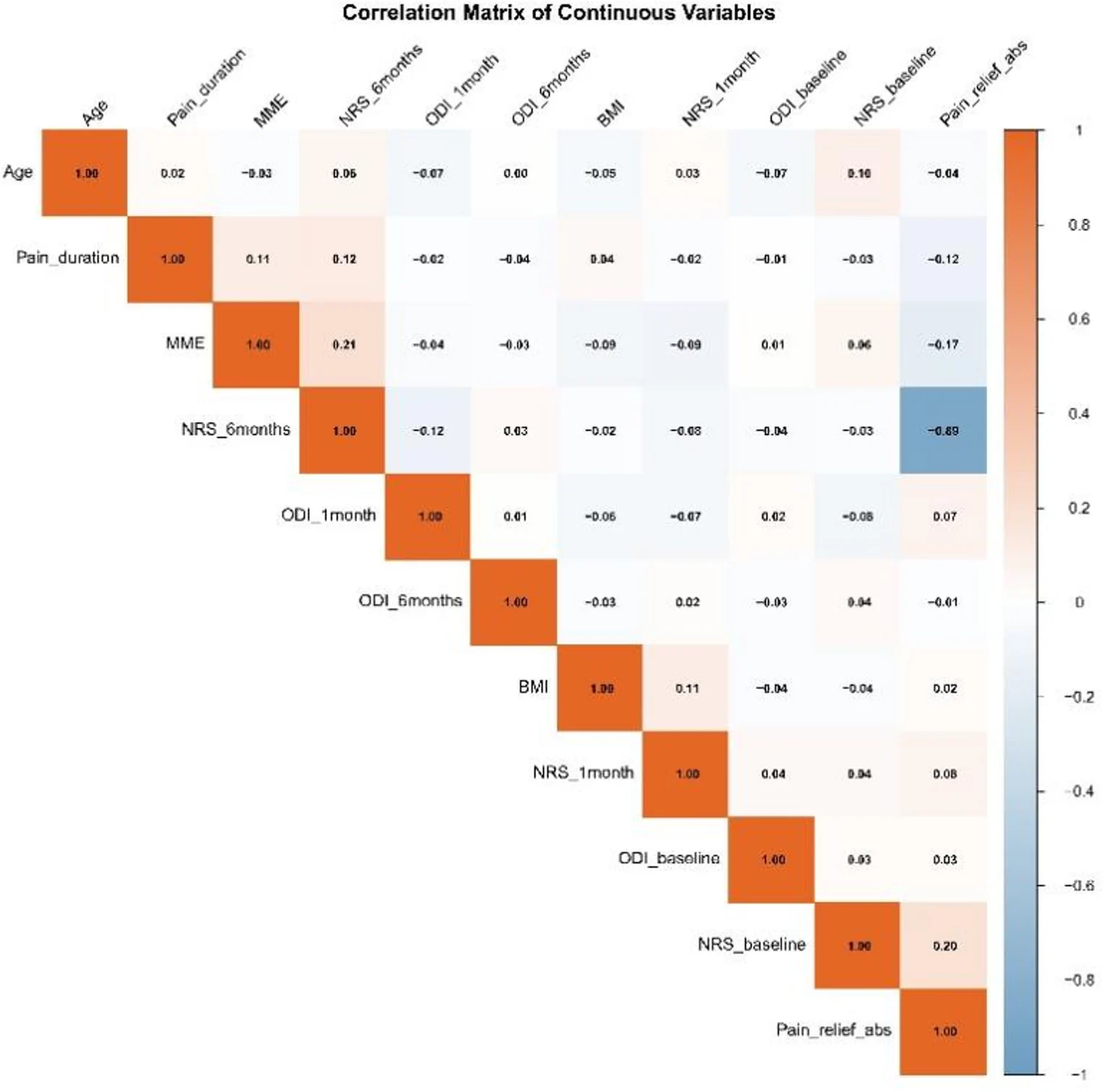
Correlation matrix of continuous clinical variables

A comprehensive Pearson correlation analysis was conducted to evaluate potential linear associations among clinical, demographic and outcome-related continuous variables (Fig. 2). Overall, the correlation structure revealed weak relationships, with most coefficients clustering near zero (range between − 0.10 and + 0.10), indicating relative independence between predictors. The only prominent association in the matrix was observed between the pain relief percentage and NRS at 6months (*r* = − 0.890), indicating a very strong inverse correlation; as expected, greater pain relief corresponds to lower NRS values at 6 months.

**Fig. 3:**
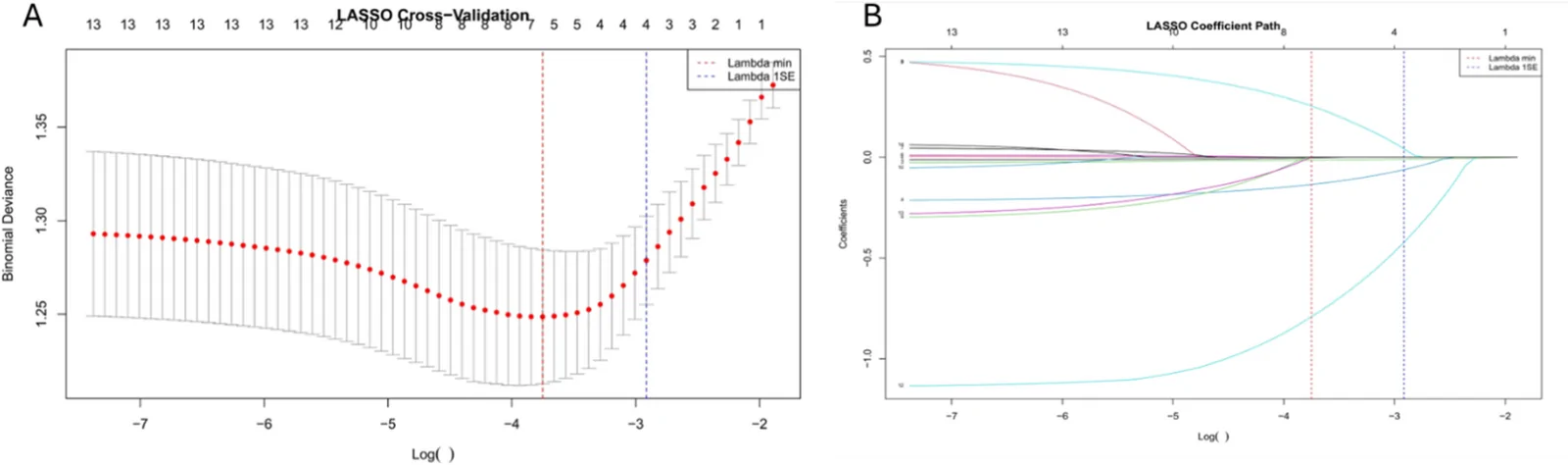
LASSO model tuning and coefficient shrinkage. **A** Tenfold cross-validation curve for the LASSO logistic regression model illustrating binomial deviance across the log-λ sequence. The red dotted line marks the optimal penalty value (λ_min = 0.02349), which achieved the lowest cross-validated deviance. The blue dotted line represents the more conservative (λ_1SE = 0.05426), selected for model parsimony. **B** LASSO coefficient trajectories for all predictors as a function of log-λ. As penalization increases, coefficients shrink progressively toward zero. At λ_1SE, four variables retained non-zero coefficients (MME, pain duration, baseline NRS and fusion surgery), defining the final reduced feature set

The penalized LASSO regression analysis identified four variables with non-zero regression coefficients:

MME, pain duration, baseline NRS, and surgery type (Fusion), (Fig. 3). All remaining variables were shrunk to zero after penalization.

## Multivariable Logistic Regression Model

The LASSO-selected variables for the multivariable logistic regression included the following variables: MME, pain duration, baseline NRS and surgery type. The LASSO coefficients are reported in Table 2. The regression equation was: $\:\mathrm{logit}\left(p\right)=-2.459-0.019\cdot\:\mathrm{MME}-0.203\cdot\;\mathrm{Pain}\;\mathrm{Duration}+0.443\cdot\:\mathrm{NRS}\_\mathrm{baseline}-1.157\cdot\;\mathrm{Surgery}\; \mathrm{Type}\;\mathrm{(Fusion)}$. Where “p” is the probability of achieving a positive clinical outcome. Among the predictors, higher pre-treatment MME emerged as a strong negative determinant of clinical success (OR = 0.981; *p* < 0.001). Longer pain duration similarly reduced the likelihood of treatment response (OR = 0.816; *p* = 0.0079). In contrast, higher baseline NRS scores were associated with a greater probability of improvement (OR = 1.558; *p* = 0.0134), suggesting that patients with more severe initial pain may derive more benefit. Surgical history also influenced outcomes: fusion or decompression procedures were significantly associated with poorer results (OR = 0.315; *p* = 0.004), whereas prior discectomy showed no measurable effect on treatment efficacy (*p* = 0.973), (Table 2).

**Table 2:** Predictors selected by LASSO and their multivariable logistic regression performance

Predictor	LASSO Coefficient (β)	Estimate (β)	OR	95% CI	*p*-value
MME	-0.0104	-0.0193	0.981	0.973–0.989	< 0.001
Pain duration	-0.0619	-0.2030	0.816	0.701–0.947	0.0079
NRS_baseline	+ 0.0413	0.4433	1.558	1.103–2.231	0.0134
Surgery: Discectomy Fusion/Decompression	- -0.4222	0.0120 -1.1566	1.012 0.315	0.508–2.018 0.137–0.670	0.9726 0.0040

The model demonstrated good calibration (Hosmer–Lemeshow *p* = 0.454) and multicollinearity was minimal (all VIF < 1.1), ensuring stable coefficient estimation.

## ROC Curve Analysis

**Fig. 4:**
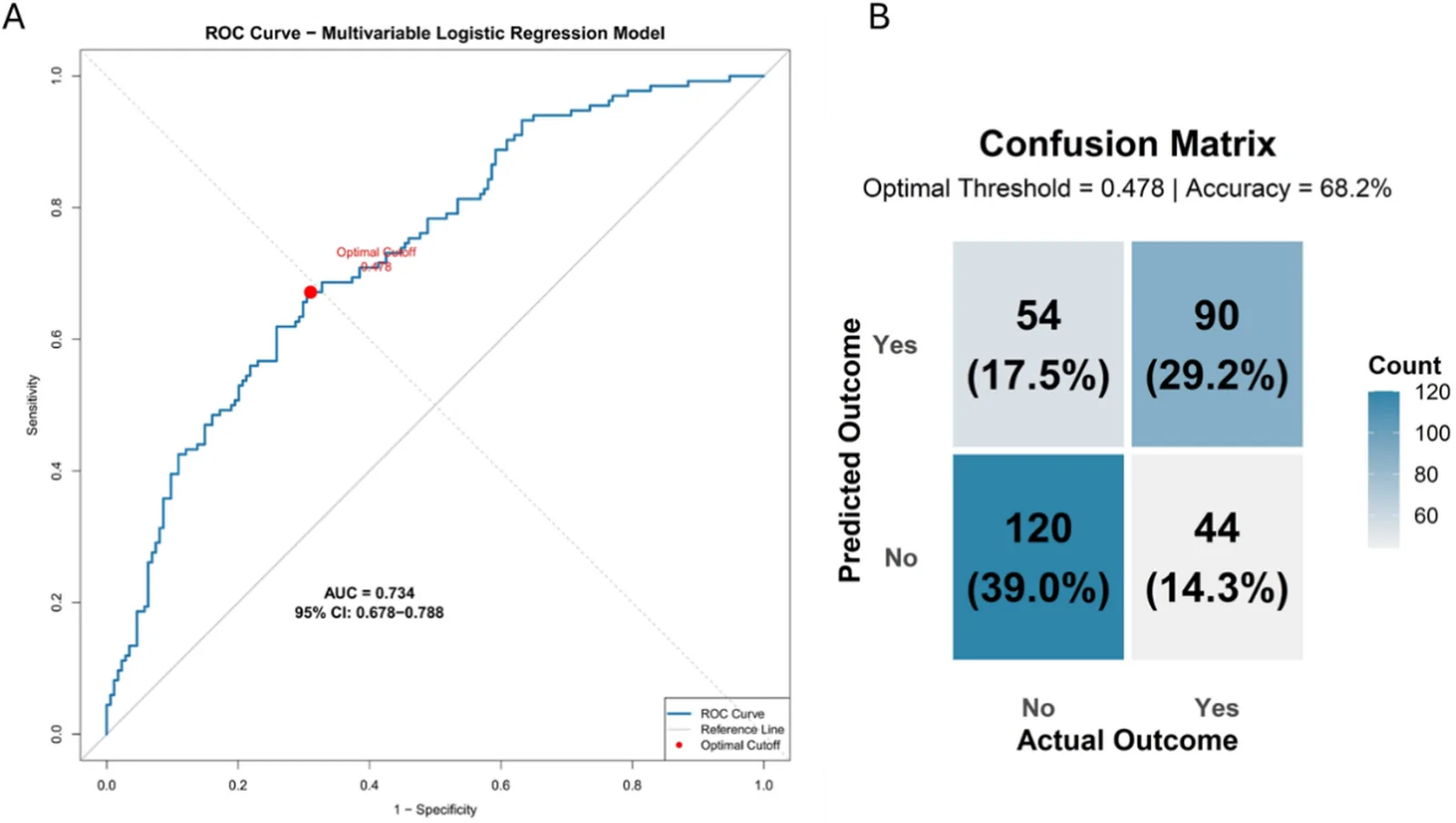
Model discrimination and classification performance. **A** The receiver operating characteristic (ROC) curve illustrates the discriminative ability of the multivariable logistic regression model to predict a positive clinical outcome. **B** Confusion matrix displays classification performance at this optimal cutoff. The model correctly identified 54 true positives (17.5%) and 120 true negatives (39.0%), with an overall accuracy of 68.2%, a positive predictive value (PPV) of 37.5%, and a negative predictive value (NPV) of 73.2%

The multivariable logistic regression model demonstrated satisfactory discriminative performance, with an AUC of 0.734 (95% CI: 0.678–0.788), indicating moderate ability to distinguish between patients with and without a positive clinical outcome (Fig. 4A). Using Youden’s index, the optimal probability cutoff was identified at 0.478, which yielded a sensitivity of 0.672 and specificity of 0.690, along with a positive predictive value of 0.625 and a negative predictive value of 0.732. At this threshold, overall classification accuracy reached 68.2%, as reflected in the confusion matrix (TN = 120, TP = 90, FN = 44, FP = 54), (Fig. 4B).

To assess model stability and potential overfitting, internal validation was performed using 10,000 bootstrap replications. The bootstrap mean AUC was 0.742 (95% CI: 0.686–0.795), closely aligned with the original model (AUC = 0.734). The estimated optimism was minimal (–0.008), resulting in an optimism-corrected AUC of 0.742, confirming good internal validity.

### Calibration Analysis

**Fig. 5:**
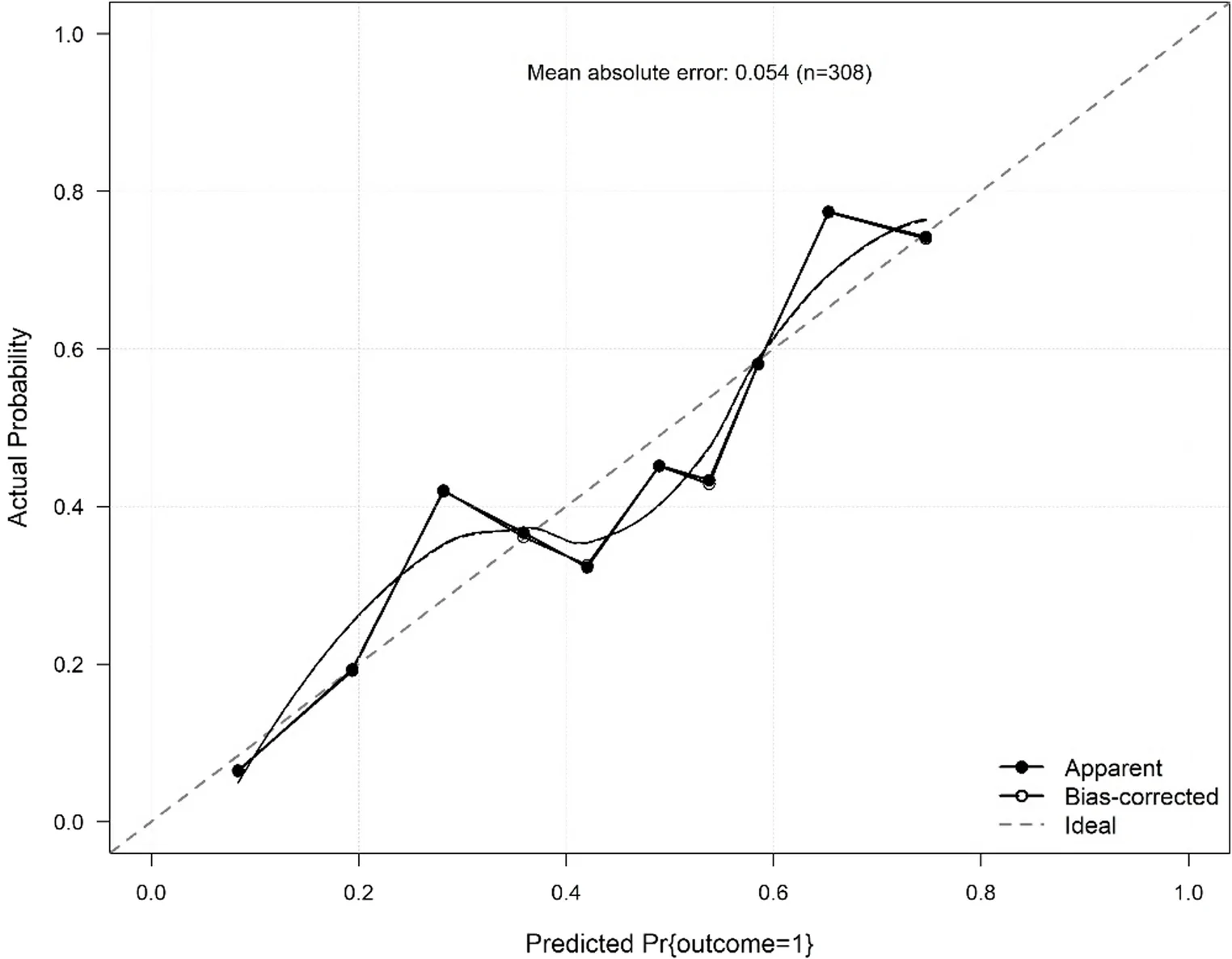
Bootstrap-corrected calibration curve for the multivariable logistic regression model. The plot shows close alignment between predicted and observed probabilities, with minimal deviation from the ideal calibration line (mean absolute error = 0.054). The bias-corrected curve indicates good model calibration and low overfitting risk

Calibration analysis demonstrated good agreement between predicted and observed outcome probabilities. The bootstrap-corrected calibration curve showed minimal deviation from the ideal reference line, with a mean absolute error of 0.054. The apparent and bias-corrected curves closely overlapped, indicating limited overfitting and strong internal calibration of the model’s probability estimates (Fig. 5).

### Nomogram Development and Prediction Formula

**Fig. 6:**
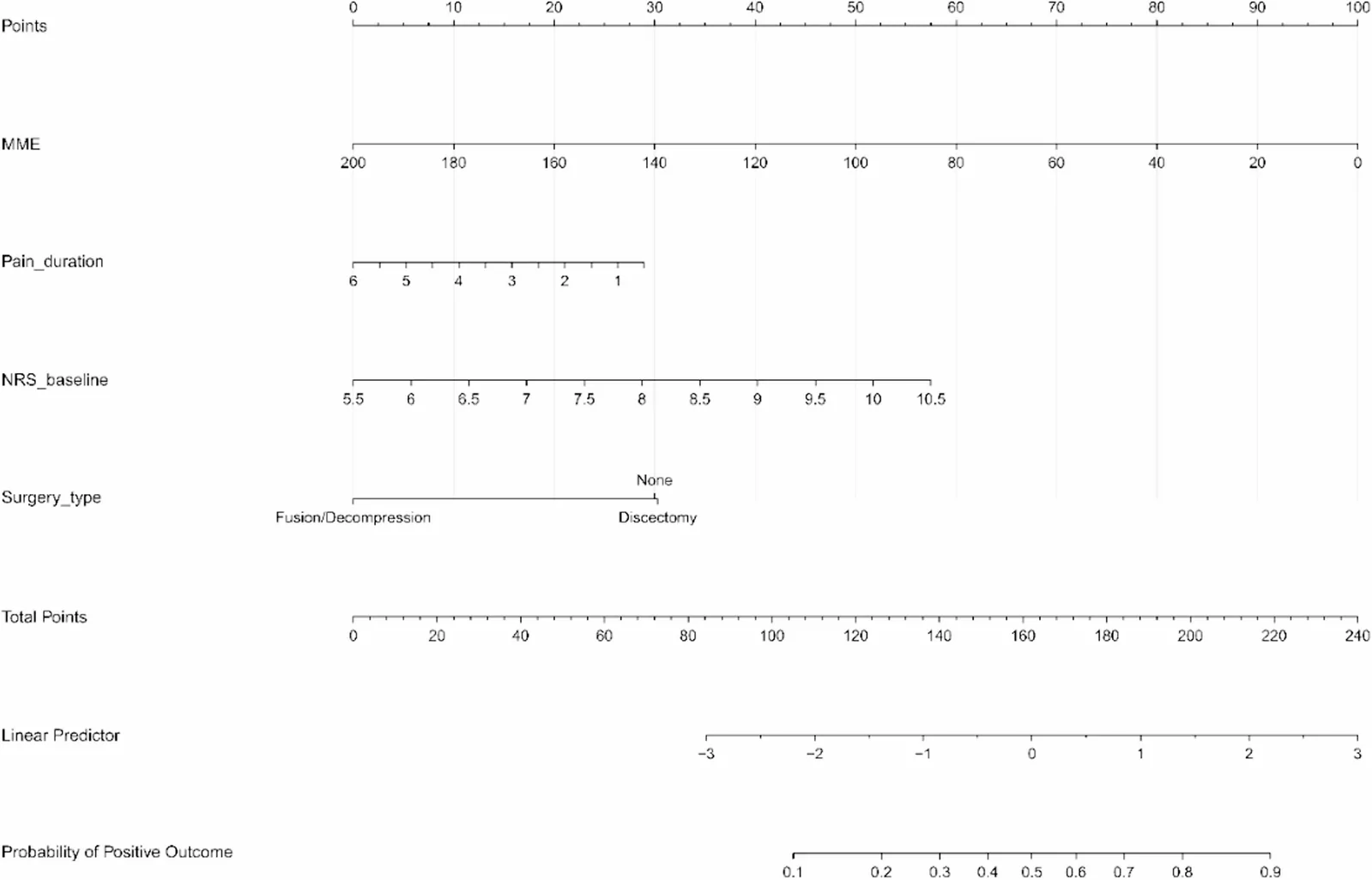
Nomogram for predicting positive outcome after lumbar DRG-PRF. The nomogram presents the multivariable logistic regression model used to estimate the probability of achieving a positive clinical outcome following lumbar DRG-PRF. Each predictor is assigned a corresponding point value on the uppermost scale; summing these values produces a Total Points score, which maps onto a linear predictor and finally to an estimated probability of a positive outcome

Using the final multivariable logistic regression model, a clinical nomogram was developed to provide an individualized estimate of the probability of achieving a positive clinical outcome after lumbar DRG-PRF (Fig. 6). The model incorporated the four LASSO-selected predictors and the resulted nomogram translates regression coefficients into a user-friendly visual scoring tool, enabling clinicians to sum predictor-specific point values and obtain an estimated probability of treatment success. Practical instructions for the use of this nomogram are provided in the Supplementary Material.

To support reproducible clinical application, the corresponding mathematical model was also derived. The final prediction equation is:

$$\begin{array}{l} \:\mathrm{log-odds}=-2.4591{\hspace{0.25em}\hspace{0.05em}}-{\hspace{0.25em}\hspace{0.05em}}0.0193\left(\mathrm{MME}\right){\hspace{0.25em}\hspace{0.05em}}-{\hspace{0.25em}\hspace{0.05em}}0.2030\left(\mathrm{Pain\:Duration}\right){\hspace{0.25em}\hspace{0.05em}}\\+{\hspace{0.25em}\hspace{0.05em}}0.4433\left({\mathrm{NRS}}_{\mathrm{baseline}}\right){\hspace{0.25em}\hspace{0.05em}}+{\hspace{0.25em}\hspace{0.05em}}0.0120\left(\mathrm{Discectomy}\right){\hspace{0.25em}\hspace{0.05em}}-{\hspace{0.25em}\hspace{0.05em}}1.1566\left(\mathrm{Fusion/Decompression}\right) \end{array}$$

The estimated probability of a positive outcome is computed as:

$$\:P\left(\mathrm{Positive\:Outcome}\right)=\frac{1}{1+{e}^{-\left(\mathrm{log-odds}\right)}}$$

As a practical illustration of how the model can be applied at the bedside, consider a patient with the following characteristics: baseline NRS = 8, pain duration = 3 years, daily opioid consumption = 10 MME and no history of lumbar surgery (neither discectomy nor fusion/decompression). For this profile, the linear predictor (log-odds) is calculated as:

$$\:\mathrm{log-odds}=-2.4591-0.0193\times\:10-0.2030\times\:3+0.4433\times\:8$$ that is,

$$\:\mathrm{log-odds}=-2.4591-0.1930-0.6090+3.5464\approx\:0.285.$$

The corresponding probability of achieving a positive outcome is then:

$$\:P\left(\mathrm{Positive\:Outcome}\right)=\frac{1}{1+{e}^{-0.285}}\approx\:0.57,$$

indicating an estimated 57% chance of treatment success for this patient.

## Discussion

This study developed and internally validated a clinically applicable prediction model to identify patients most likely to benefit from lumbar DRG-PRF for chronic radicular pain. Using LASSO-based feature selection, four independent predictors of treatment success at 6 months were identified: baseline pain intensity, opioid consumption (MME), pain duration and type of previous lumbar surgery. The resulting multivariable model demonstrated moderate discriminative ability with good calibration and robust internal validation. Higher opioid use, longer pain duration, and prior fusion or decompression surgery were associated with a reduced likelihood of success, whereas higher baseline pain intensity paradoxically predicted greater improvement. The translation of these findings into a nomogram offers a practical tool for individualized patient selection and pre-procedural counseling.

Our observed treatment response rates align with the heterogeneous outcomes reported in the existing literature on PRF for lumbar radicular pain. In fact, recent systematic reviews and meta-analyses have reported success rates ranging from 32% to 87% depending on patient selection criteria, outcome definitions, and follow-up duration, underscoring substantial variability in treatment response [21, 22]. In our cohort, 43.5% of patients achieved the primary outcome of ≥ 50% pain reduction at 6 months, which falls within the mid-range of published estimates and is comparable to the 46% responder rate reported in our recently published propensity-matched analysis [19]. The magnitude of pain reduction observed in our study (overall NRS decrease of 3.48 points) is clinically meaningful and exceeds the commonly accepted minimal clinically important difference of 2 points for chronic pain conditions [23–25].

Importantly, our data demonstrates sustained analgesic benefits of DRG-PRF up to the 6-month follow-up. These findings are consistent with previous studies with extended follow-up, which have documented persistent pain relief lasting up to 6–12 months after the procedure [26, 27]. The emergence of baseline opioid consumption as the strongest negative predictor in our model carries significant clinical implications. In fact, each 1 mg increase in daily MME was associated with a 2% reduction in odds of treatment success, suggesting a dose-dependent relationship between chronic opioid exposure and diminished PRF efficacy. This finding aligns with a growing body of evidence documenting the adverse influence of opioid therapy on interventional pain treatment outcomes across multiple modalities and anatomical targets [28–33].

From a clinical point of view, our findings suggest that efforts to taper or optimize opioid therapy prior to DRG-PRF intervention may enhance treatment success rates. In fact, the 2022 CDC Clinical Practice Guideline for Prescribing Opioids emphasizes the importance of multimodal pain management, incorporating non-opioid pharmacotherapy and non-pharmacological approaches, particularly for chronic non-cancer pain [34]. Interventional pain specialists should consider coordinated opioid dose reduction strategies in conjunction with PRF treatment, recognizing that lower opioid use may not only improve procedural outcomes but also mitigate long-term risks of opioid-related adverse events [35, 36].

Our model identified previous lumbar fusion or decompression surgery as a strong negative predictor of DRG-PRF success (OR = 0.315), whereas prior discectomy alone showed no significant effect. This differential impact of surgery type has important implications for patient counseling and treatment planning. The unfavorable prognosis associated with prior fusion procedures is consistent with the well-characterized phenomenon of failed back surgery syndrome (FBSS), recently reclassified as persistent spinal pain syndrome type 2 (PSPS-T2) in the International Classification of Diseases, 11th Revision [37]. Patients with FBSS/PSPS-T2 represent a particularly challenging population characterized by complex pain mechanisms, psychological comorbidities, and altered biomechanics that collectively contribute to treatment resistance [38, 39]. The pathophysiology of persistent pain following fusion surgery is multifactorial and may include epidural fibrosis causing nerve root tethering and mechanical irritation, adjacent segment disease due to altered load distribution, pseudarthrosis with persistent instability, retained foreign bodies or hardware complications, and central sensitization resulting from prolonged nociceptive input [40]. Each of these mechanisms may attenuate the effectiveness of PRF, which primarily targets peripheral and segmental pain processing at the DRG level but may be insufficient to address centralized pain states or structural biomechanical derangements. Consequently, it can be hypothesized that patients with a history of lumbar fusion or decompression surgery may obtain greater benefit from alternative therapeutic approaches, such as epidural PRF [41], epidurolysis [42] or epiduroscopy [43], rather than DRG-PRF.

Recent retrospective analyses have corroborated the negative prognostic significance of previous spine surgery for PRF outcomes. Jandura et al. [44] reported that prior lumbar surgery decreased the therapeutic efficacy of DRG-PRF in patients with chronic lumbosacral radicular pain, though the mechanism underlying this association remains incompletely understood.

In contrast, another study [45] did not identify a statistically significant association between previous surgery and PRF outcomes, although a nonsignificant trend was observed (*p* = 0.09). However, several methodological differences should be considered when interpreting these findings. First, the outcome assessment was performed at earlier time points (10 days, 1 month, and 3 months) compared with the 6-month follow-up used in our study. Second, PRF was combined with transforaminal steroid injection, precluding evaluation of the isolated effect of PRF alone. Third, the PRF protocol differed substantially, as a shorter treatment duration was applied (45 V, 42 °C for 120 s). Treatment duration appears to be clinically relevant, as recent randomized controlled trials have demonstrated superior outcomes with longer PRF application times, particularly 6-minute protocols [46]. Finally, previous surgery was classified dichotomously without distinguishing between surgical types. This distinction is clinically important, as different surgical procedures are associated with varying degrees of epidural fibrosis, which may differentially influence PRF efficacy [47, 48]. Moreover, the role of previous surgery is multifactorial: the complexity of post-surgical anatomy, including scar tissue formation and altered neural relationships, may compromise needle placement accuracy and reduce the effective delivery of electromagnetic fields to the target ganglion. Furthermore, patients who have undergone multiple surgical interventions may harbor unrealistic expectations or demonstrate maladaptive illness behaviors that independently influence perceived treatment outcomes [49]. In contrast, the absence of a negative association with prior discectomy in our cohort confirm that less extensive surgical interventions may not alter the neurophysiological substrate in a manner that compromises PRF efficacy. Discectomy typically addresses focal mechanical compression without extensive soft tissue dissection or biomechanical alteration, potentially explaining the preserved treatment responsiveness. Finally, this finding may be explained by the complex and multifactorial nature of pain in patients with a history of spinal surgery. In these individuals, pain is often not exclusively mediated by the DRG but may also arise from adjacent anatomical structures, including facet joints, intervertebral discs, paraspinal muscles, and scar-related changes [50]. As a result, targeting the DRG alone may be insufficient to address all relevant pain generators, which could account for the reduced effectiveness of DRG radiofrequency observed in this subgroup.

The finding that higher baseline pain intensity predicted increased likelihood of positive outcome (OR = 1.558 per 1-point NRS increase) represents a counterintuitive but potentially important observation. This paradox, wherein patients presenting with more severe initial pain demonstrate superior treatment responses, has been previously reported in the PRF literature but lacks a unified mechanistic explanation [16, 51]. Several potential mechanisms may explain this association. Patients with more severe radicular pain may have greater capacity for clinically meaningful improvement. Moreover, patient motivation or expectancy effects may also contribute. Evidence in the literature remains limited and this finding requires external validation. Consequently, baseline pain intensity should be interpreted in conjunction with other prognostic factors rather than in isolation, to guide individualized patient selection.

Longer pain duration emerged as another independent negative predictor in our model, with each additional year of symptoms associated with an 18% reduction in odds of treatment success (OR = 0.816). This temporal dimension of prognosis aligns with established principles of pain chronification, wherein prolonged nociceptive input induces progressive neuroplastic changes within peripheral, spinal, and supraspinal pain processing networks that render chronic pain increasingly refractory to intervention [52]. Clinical studies across multiple pain conditions have documented declining treatment efficacy with increasing symptom duration. In the context of lumbar radicular pain specifically, many articles have suggested that patients treated within the first year of symptom onset demonstrate superior responses to those with multi-year symptomatology [32, 53–55].

The moderate discriminative ability of our model (AUC = 0.734) compares favorably with published prediction models for other interventional pain procedures such as continuous radiofrequency ablation of facet joints [56]. To our knowledge, this represents the first validated prediction model specifically developed for PRF treatment of lumbar DRG. However, the sensitivity and specificity of the model indicate that approximately one-third of patients may be misclassified. Accordingly, several considerations must be addressed before clinical implementation. First, external validation in independent cohorts is required. Although internal bootstrap validation confirmed the statistical robustness of the model within our dataset, variations in geography, patient demographics, and clinical practice patterns may limit its generalizability. Second, the model does not incorporate procedural factors such as the specific equipment used (e.g., needle type, active tip length and PRF generator), operator experience, or detailed PRF parameters (including voltage, duration, impedance, pulse width, and frequency), all of which may independently affect treatment outcomes. Third, the outcome definition was limited to pain reduction and did not include broader measures of treatment success, such as functional improvement, quality of life, disability scores, or patient satisfaction, which are increasingly recognized as essential endpoints in pain management. Within these limitations, the proposed model offers several clinically meaningful applications. It may support patient selection and prioritization by identifying individuals most likely to benefit from DRG-PRF, while guiding those with unfavorable prognostic profiles toward alternative therapeutic strategies. In addition, the model can facilitate informed consent and expectation management by providing personalized, probabilistic estimates of treatment success, thereby enhancing shared decision-making. From a health-system perspective, it may assist in resource allocation by optimizing the use of limited procedural capacity. Finally, the model may serve as a stratification tool for clinical research, enabling the enrollment of prognostically homogeneous cohorts and improving the design of comparative effectiveness studies. The heterogeneity of responses observed in our cohort likely reflects interindividual differences in underlying pain mechanisms, underscoring the potential value of future models integrating clinical, imaging and neurobiological markers to further refine patient selection.

### Limitations

This study has several limitations that should be acknowledged. First, its retrospective observational design entails inherent risks of selection bias and residual confounding. Although we adjusted for a broad set of clinical variables, unmeasured factors such as psychological comorbidities, socioeconomic stressors, or genetic predisposition may have influenced outcomes. Prospective studies with standardized data collection would help mitigate these limitations. Second, the single-center setting may limit generalizability, as patient characteristics, referral patterns, and procedural practices can vary across institutions and healthcare systems. External validation in geographically and demographically diverse cohorts is therefore required before routine clinical adoption of the nomogram. Third, the definition of positive outcome relied exclusively on a ≥ 50% reduction in NRS at 6 months, which does not fully capture the multidimensional benefits of treatment. Some patients may have achieved meaningful improvements in function, medication use, or quality of life despite not meeting this pain threshold. Future studies should incorporate composite endpoints integrating pain, disability, patient-reported outcomes, and adverse events. Several potentially relevant predictors were not systematically collected, including psychological factors (e.g., depression and pain catastrophizing), imaging characteristics, procedural variables, operator experience, and concomitant therapies. Psychological comorbidities, in particular, are known negative prognostic factors for chronic pain interventions [57, 58], and their omission represents an important limitation. Medication exposure was derived from medical records rather than objective dispensing data, which may have introduced imprecision in opioid dose estimation. Moreover, opioid use disorder and aberrant medication behaviors, both associated with poorer outcomes [59], were not formally assessed.

The 6-month follow-up duration, while clinically relevant and consistent with most PRF literature, does not capture very long-term outcomes. Some patients may experience delayed treatment failure beyond 6 months, while others may continue to derive benefit at 1–2 years post-procedure. Extended follow-up studies are needed to define the durability of PRF effects and to determine whether early predictors of 6-month outcomes also predict sustained long-term benefit.

Finally, our study examined DRG-PRF as a standalone intervention without accounting for adjunctive treatments such as transforaminal epidural steroid injection, which is sometimes administered concurrently with PRF. In fact, in our previously published paper [19] and as confirmed by other authors [60], we found no additional benefit of steroid injection when combined with PRF alone.

### Future Research Directions, Clinical Implications and Recommendations

Several future research priorities arise from our findings. As previously reported, external validation of the prediction model in independent and geographically diverse cohorts is essential to confirm generalizability and guide potential recalibration. Further studies should also optimize PRF procedural parameters, including voltage, pulse duration and total treatment time. Cost-effectiveness analyses are needed to determine whether risk-stratified PRF improves healthcare efficiency. Finally, interventional strategies targeting modifiable poor prognostic factors, such as opioid use or psychological distress, may further enhance treatment response and advance personalized pain management. Clinically, routine preprocedural assessment of baseline pain intensity, opioid use, pain duration and surgical history is recommended to support individualized risk estimation and informed consent using the proposed nomogram. High opioid consumption and prior fusion or decompression surgery warrant careful counseling and consideration of alternative or adjunctive strategies, whereas prior discectomy alone should not preclude DRG-PRF. Earlier referral may improve outcomes and patients with severe pain but otherwise favorable profiles may be prioritized in resource-limited settings. Importantly, unfavorable predictions should be viewed as markers of guarded prognosis rather than absolute contraindications, reinforcing the role of shared decision-making and systematic outcome monitoring to refine future models.

## Conclusion

This study developed and internally validated a clinical prediction model to identify patients most likely to benefit from lumbar DRG-PRF. Higher opioid consumption, longer pain duration, and prior lumbar fusion surgery were associated with poorer outcomes, whereas higher baseline pain intensity predicted greater likelihood of response. The model showed moderate discriminative performance with good calibration and minimal optimism on internal validation, and its translation into a practical nomogram enables individualized risk stratification, informed consent, and prognostic counseling. Clinically meaningful improvements in pain were observed, with 43.5% of patients achieving ≥ 50% pain reduction at 6 months. Notably, surgical history demonstrated differential prognostic value, as prior fusion, but not discectomy, was associated with reduced treatment success, underscoring the importance of surgical subtype rather than prior surgery per se. Implementation of this model may improve patient selection, optimize resource utilization, and support shared decision-making. External validation and prospective risk-stratified studies are needed to confirm generalizability and to explore whether pre-procedural optimization, particularly opioid reduction and psychological support, can further enhance outcomes.

## Key References

- Park J-H, Jang JN, Park S, Choi Y-S, Ryu DK, Miah R, et al. Pulsed radiofrequency of lumbar dorsal root ganglion versus epidural neuroplasty for lumbar radicular pain: a systematic review and network meta-analysis. Reg Anesth Pain Med. 2026;51(6):621–629. 10.1136/rapm-2025-106723.
  - ○ This recent systematic review and network meta-analysis synthesized 14 randomized controlled trials including 1,229 patients and represents one of the most comprehensive contemporary evaluations of DRG-PRF for lumbar radicular pain. Although the certainty of evidence was generally low to very low, it provides an important comparative framework for positioning DRG-PRF relative to epidural neuroplasty and other interventional strategies and highlights the persistent need for improved patient selection and high-quality comparative trials.
- Leoni MLG, Micheli F, Abbott DM, Cascella M, Varrassi G, Sansone P, et al. Transforaminal Steroid Injection After Dorsal Root Ganglion Pulsed Radiofrequency (DRG-PRF): Impact on Pain Intensity and Disability. Pain Ther. 2024;13:1271–1285. 10.1007/s40122-024-00639-w.
  - ○ This propensity score-matched study demonstrated clinically meaningful improvements in pain and disability following lumbar DRG-PRF and found no additional benefit from immediate transforaminal epidural steroid injection. The findings support the clinical effectiveness of DRG-PRF as an independent intervention and provide important context for interpreting outcomes in the present cohort.
- Jang JN, Park S, Park J-H, Choi Y-S, Park S. Efficacy of pulsed radiofrequency treatment duration to the lumbar dorsal root ganglion in lumbar radicular pain: a double-blind randomized controlled trial. Reg Anesth Pain Med. 2025. 10.1136/rapm-2025-106908.
  - ○ This double-blind randomized controlled trial directly compared different DRG-PRF treatment durations and showed that a 6-minute application produced the most consistent improvements in pain and function, with possible additional benefits from an 8-minute protocol in selected patients. The study is particularly relevant to the present work because it provides prospective evidence supporting the clinical importance of procedural parameters.
- Yildiz G, Goksu H, Akcaboy EY, Celik S, Ayhan MY, Kaya SS. Does transforaminal epidural steroid injection added to dorsal root ganglion pulsed radiofrequency treatment increase efficacy? Turk J Phys Med Rehabil. 2024;70:390–396. 10.5606/tftrd.2024.13479.
  - ○ This contemporary clinical study further examined whether transforaminal epidural steroid injection provides additional benefit when combined with DRG-PRF. Together with other recent comparative evidence, it contributes to the ongoing discussion regarding the necessity of adjunctive steroid administration and supports refinement of procedural protocols for lumbar radicular pain.

## Supplementary Information

Below is the link to the electronic supplementary material.

Supplementary Material 1

## Data Availability

Data are available upon reasonable request to the corresponding author.

## Abbreviations

AUC: Area Under the Curve
BMI: Body Mass Index
CDC: Centers for Disease Control and Prevention
CI: Confidence Interval
DRG: Dorsal Root Ganglion
DRG-PRF: Dorsal Root Ganglion Pulsed Radiofrequency
FBSS: Failed Back Surgery Syndrome
FN: False Negative
FP: False Positive
ICD-11: International Classification of Diseases, 11th Revision
IQR: Interquartile Range
LASSO: Least Absolute Shrinkage and Selection Operator
MME: Morphine Milligram Equivalents
NPV: Negative Predictive Value
NRS: Numerical Rating Scale
OR: Odds Ratio
PPV: Positive Predictive Value
PRF: Pulsed Radiofrequency
PSPS-T2: Persistent Spinal Pain Syndrome Type 2
ROC: Receiver Operating Characteristic
SD: Standard Deviation
TN: True Negative
TP: True Positive
TRIPOD: Transparent Reporting of a multivariable prediction model for Individual Prognosis Or Diagnosis
VIF: Variance Inflation Factor
