# Supplementary material for: Predictors of Treatment Success Following Pulsed Radiofrequency of the Lumbar Dorsal Root Ganglion: A Multivariable Prediction Model with Clinical Nomogram
Source: Curr Pain Headache Rep. 2026 Aug 24;30(1):110. doi: 10.1007/s11916-026-01544-x (PMC13503403; doi:10.1007/s11916-026-01544-x)
Supplement: Supplementary file 1 [file 11916_2026_1544_MOESM1_ESM.docx]

**Supplementary material**

Practical application of the clinical nomogram

The clinical nomogram (Fig. 6) provides a user-friendly graphical tool that translates the multivariable prediction model into point-of-care risk assessment without requiring complex mathematical calculations. The nomogram consists of several components: individual predictor scales showing the range of possible values for each variable; a points scale at the top that converts each predictor value to a numerical score; a total points scale that sums the individual scores; and a probability scale at the bottom that converts the total points into the predicted probability of achieving ≥50% pain reduction at 6 months.

To use the nomogram in clinical practice, clinicians follow a systematic five-step process:

**Step 1**: For each of the four predictors, locate the patient's value on the corresponding axis and draw a vertical line upward to intersect the "Points" scale at the top of the nomogram. Record the point value for each predictor.

**Step 2**: Sum the individual point values to calculate the total points. This aggregate score reflects the combined prognostic influence of all four predictors, weighted according to their relative importance in the model.

**Step 3**: Locate the total points on the "Total Points" axis and draw a vertical line downward to intersect the "Probability" scale at the bottom of the nomogram.

**Step 4**: Read the predicted probability directly from the probability scale. This value represents the estimated likelihood (expressed as a percentage from 0% to 100%) that the patient will achieve the primary outcome of ≥50% pain reduction at 6-month follow-up.

**Step 5**: Integrate the probability estimate into a shared decision-making discussion with the patient, considering individual values, preferences, alternative treatment options, and clinical judgment.

Clinical Example 1: Favorable prognosis profile

Consider a patient presenting with the following characteristics:

- Baseline NRS pain score: 8/10

- Pain duration: 2 years

- Daily opioid consumption: 0 MME (opioid-naive)

- Previous spine surgery: None

Using the nomogram:

- Baseline NRS = 8 → approximately 15 points

- Pain duration = 2 years → approximately 20 points

- Opioid dose = 0 MME → approximately 25 points

- No previous surgery (reference category) → 0 points

- Total points = 60

Projecting 60 total points down to the probability scale yields an estimated probability of 75% for achieving treatment success. This high probability indicates that this patient represents an ideal candidate for PRF treatment.

Clinical Example 2: Moderate prognosis profile

Consider a patient with:

- Baseline NRS = 8/10

- Pain duration = 3 years

- Daily opioid consumption = 10 MME

- Previous spine surgery: None

Using the nomogram:

- Baseline NRS = 8 → approximately 12 points

- Pain duration = 3 years → approximately 18 points

- Opioid dose = 10 MME → approximately 22 points

- No previous surgery → 0 points

- Total points = 52

Projecting 52 total points to the probability scale yields approximately 57% probability of treatment success.

Clinical Example 3: Unfavorable prognosis profile

Consider a patient presenting with multiple poor prognostic features:

- Baseline NRS = 7

- Pain duration = 5 years

- Daily opioid consumption = 60 MME

- Previous lumbar fusion surgery

Using the nomogram:

- Baseline NRS = 7 → approximately 8 points

- Pain duration = 5 years → approximately 10 points

- Opioid dose = 60 MME → approximately 8 points

- Previous fusion → approximately −20 points (negative contribution)

- Total points = 6

Projecting 6 total points to the probability scale yields an estimated probability of approximately 20% for treatment success.

Clinical Example 4: Effect of opioid reduction

To illustrate the prognostic impact of modifiable risk factors, consider how pre-procedural opioid tapering might influence predicted outcomes.

A patient initially presents with:

- Baseline NRS = 8

- Pain duration = 2 years

- Daily opioid consumption = 40 MME

- No previous surgery

Initial nomogram calculation:

- NRS = 8 → 15 points

- Duration = 2 years → 20 points

- Opioids = 40 MME → 15 points

- No surgery → 0 points

- Total = 50 points → ~55% probability

After a structured tapering program reduces opioid consumption to 10 MME:

- NRS = 8 → 15 points

- Duration = 2.25 years → 19 points

- Opioids = 10 MME → 22 points

- No surgery → 0 points

- Total = 56 points → ~65% probability

This 10-percentage-point improvement in predicted probability (from 55% to 65%) demonstrates the tangible benefit of optimizing modifiable risk factors prior to intervention.

Importantly, the nomogram predictions should be presented probabilistically rather than deterministically.

The clinical significance of different probability thresholds merits consideration. While no universally accepted cutoff defines "good" versus "poor" prognosis, the following framework may guide clinical decision-making:

- Probability ≥70%: Favorable prognosis; PRF represents a reasonable first-line interventional option

- Probability 50-70%: Moderate prognosis; PRF is a viable option but alternatives should be discussed

- Probability 30-50%: Guarded prognosis; consider more intensive pre-procedural optimization or alternative therapies

- Probability <30%: Unfavorable prognosis; carefully weigh risks and benefits, ensure realistic expectations, explore alternative treatments

These thresholds are not absolute contraindications but rather guide shared decision-making discussions.
